# Relation of fasting and postprandial and plasma glucose with hemoglobinA1c in diabetics

**DOI:** 10.4103/0973-3930.60002

**Published:** 2010

**Authors:** Shahram Haddadinezhad, Nargess Ghazaleh

**Affiliations:** Department of Endocrinology and Metabolism, Hamadan University of Medical sciences, Hamadan, Iran

**Keywords:** Diabetes mellitus, fasting plasma glucose postprandial plasma glucose, hemoglobinA1c

## Abstract

**Context:**

Control of plasma glucose could prevent the progression of most of the complications of diabetes and hemoglobinA1c (HbA1c) is the most important criterion controlling these long-term complications.

**Aims:**

This study was performed to assess the effect of fasting plasma glucose (FPG) and two- hour postprandial plasma glucose (2hpp) levels on HbA1c.

**Materials and Methods:**

In this descriptive, cross-sectional study; 300 patients were enrolled, assessed, and followed up at the clinic of the Diabetic Center of the University of Medical Science, Hamadan, Iran. All studied patients were diagnosed type 1 or 2 diabetes mellitus. Sampling was performed; we assessed FPG and 2hpp plasma glucose at baseline and at every two weeks to one month-as needed. HbA1c was assessed at the end of study. Results were analyzed by Pierson Covariance and Multiple Regression methods.

**Results:**

The mean plasma glucose in three groups of HbA1c (good to fair) were 148.5 ± 56.80 mg/dl at fasting, and 199.70 ± 53.01 mg/dl at two hours after breakfast (2hpp) and mean concentration of HbA1c were 8.41 ± 1.1 %. The plasma glucose level and HbA1c were 0.312 for fasting and, 0.416 for 2hpp at covariant value.

**Conclusion:**

The postprandial (after breakfast) plasma glucose has closer association to glycosylated hemoglobin than fasting plasma glucose, therefore evaluating postprandial plasma glucose should be our focus.

## Introduction

Diabetes, the most common metabolic disease, is associated with major micro and macrovascular complications.[1] Many studies demonstrate that controlling plasma glucose level could prevent the progression of these complications, especially microvascular disease.[2–5] Because the fluctuations of plasma glucose level do not lend to easy analysis, we can use the hemoglobinA1c (HbA1c), which reflects the mean plasma glucose, in the last eight to 12 weeks.[6]

Many recent studies have demonstrated that elevated postprandial plasma glucose effects the diabetes complications, primarily the in macrovascular complications more severely than elevated fasting plasma glucose.[7–9] Since fluctuations of fasting plasma glucose and postprandial could affect HbA1c, this study was performed to assess the relationship of fasting plasma glucose and two-hour postprandial on HbA1c.

## Materials and Methods

The study was performed as a descriptive, cross-sectional, on 300 diabetic patients, type 1 or 2, at the Clinic of Diabetic Center, University of Medical Science, Hamadan, Iran.

Blood sampling was done every two to four weeks by the glucose oxidase method, at fasting and two-hour after breakfast (postprandial); three samples were taken on each patient in the three-month period. Mean plasma glucose was compared at fasting and postprandial separately with HbA1c level, which had been assessed at end of the study with HbA1c analyzer calibrated to give DCCT equivalent results by the chromatographicion exchange method (biosystem kit, spain).

Patients were divided into three groups according to the American Diabetic Association (ADA) guidelines regarding to the HbA1c level; HbA1c <7.5% (well controlled), 7.5% ≤ HbA1c ≤ 9% (fairly controlled), and HbA1c >9% (poorly controlled) - HbA1c less than 6% was considered in the normal range.

## Results

A total of 300 patients (173 female and 127 male) were included, with the minimum age of 12 years and maximum of 67 years (there was no statistical difference in glycemic status between 3 groups of males and females). Two hundred and thirty seven patients were diagnosed as diabetes mellitus type 2, and 63 had type 1 diabetes. Out of 237 type 2 diabetic patients, 53 patients were on insulin therapy and the others (184 patients) were on treatment with oral medications [Table 1].

**Table 1:** Changes in variables, during the study

Characteristic	1^st^ group	2^nd^ group	3^rd^ group	Total
Female	50	62	57	169
Male	35	50	46	131
DM1^1^	17	25	21	63
DM2	69	90	78	237
Insulin therapy^2^	32	46	38	116
Antidiabetic therapy^3^	55	68	61	184
Total	85	112	103	300
FPG (mg/dl)†	121.7 ± 28.2	152.4 ± 64.3	175.3 ± 21.4	
FPG(−t_1_DM)†	120.1 ± 27.1	150.1 ± 60.2	178.6 ± 19.8	
FPG(−t_2_DM)†	123.3 ± 29.1	150.1 ± 62.4	178.6 ± 22.3	
PG2hpp(mg/dl)‡	163.2 ± 32.8	203.5 ± 45.2	231.7 ± 42.5	
PG2hpp(−t_1_DM)‡	161.8 ± 30.8	200.5 ± 43.1	233.4 ± 40.5	
PG2hpp(−t_2_DM)‡	164.6 ± 31.9	206.5 ± 46.3	230 ± 43.1	
HbA1c (%)††	6.93 ± 0.41	8.11 ± 0.42	10.2 ± 0.39	
HbA1c (−t_1_DM)	6.72 ± 0.4	7.95 ± 0.39	10.3 ± 0.38	
HbA1c (−t_2_DM)	7.14 ± 0.45	8.72 ± 0.41	10.1 ± 0.40	
Disease period*	8.63 ± 4.72	9.9 ± 3.58	10.9 ± 4.28	

1 Diabetes mellitus;

2 Insulin therapy;

3 Treatment with oral medications;

† Fasting plasma glucose;

‡ 2 hour postprandial plasma glucose;

†† Glycosylated hemoglobin;

* year

The mean fasting plasma glucose and two hours after breakfast plasma glucose level in all three groups-of total patients were 148.0 ± 56.8 mg/dl and 199.7 ± 53 mg/ dl- for type1 DM; 148.9 ± 51 mg/dl and 198.56 ± 52 mg/dl and for type-2 DM were 150.6 ± 48 mg/dl and 200.3 ± 47 mg/dl- respectively and the difference was statistically significant (*P* < 0.0001).

There was a linear relation, using multiple regression analyzers between HbA1c and plasma glucose level in type 1, type 2 and all patients (*P* < 0.0001).

The correlation coefficient of fasting plasma glucose on HbA1c was r = 0.315-(*P* < 0.001) for all patients; r = 0.309-(*P* < 0.001) for type-1 DM; r = 0.316-(*P* < 0.001) for type-2 DM. The correlation coefficient of 2 hour postprandial plasma glucose on HbA1c was r = 0.426-(*P* < 0.001) for all patients. (type-1 DM; r = 0.419, (*P* < 0.001) and r = 0.425, (*P* < 0.001) for type- 2 DM.)

## Discussion

Our results indicate that postprandial glucose level increased in all three groups, and has a strong relationship with the rising of HbA1c level. Increasing of HbA1c has shown more dependency with postprandial plasma glucose as compared to with fasting plasma glucose level.

According to many studies, HbA1c is the best criterion for the control of diabetes and for preventing diabetes complications.[10–13] A reduction of 1% of HbA1c could prevent 30-35% of microvascular and 14-16% of macrovascular complications.[14–16]

A number of studies have demonstrated that HbA1c has more relation to postprandial plasma glucose level, than fasting plasma glucose,[17,18] but on the contrary, Bonora *et al*., have observed that HbA1c has close relation to fasting plasma glucose and not to the postprandial plasma glucose level.[19,20]

Recent studies have suggested that postprandial plasma glucose elevation could produce severe cardiovascular system morbidity.[21–23] The UKPDS study, with its focus on fasting plasma glucose, did not show significant reduction in macrovascular complications[15] but in those studies where focus was on postprandial plasma glucose monitoring, better reduction of macrovascular complications. was seen.[7–9] The results of this study have shown that more than two- thirds of the patients are in poor glycemic control and exposed to the possibility of late-onset diabetic complications.
